# Reduction of aflatoxins during brewing of a Malawian maize‐based non‐alcoholic beverage, *thobwa*

**DOI:** 10.1002/fsn3.3266

**Published:** 2023-03-13

**Authors:** Sydney Namaumbo, Maurice Monjerezi, Aggrey Gama, Angstone Thembachako Mlangeni, Gabriella Chiutsi‐Phiri, Limbikani Matumba

**Affiliations:** ^1^ Faculty of Life Science and Natural Resources Lilongwe University of Agriculture and Natural Resources (LUANAR) Lilongwe Malawi; ^2^ Department of Chemistry and Chemical Engineering University of Malawi Zomba Malawi; ^3^ Faculty of Food and Human Sciences, LUANAR Lilongwe Malawi

**Keywords:** aflatoxin, boiling, brewing, fermenting, *Thobwa*

## Abstract

This study reports onthe effect of various production steps on levels of aflatoxins during preparation of *thobwa*, a traditional maize‐based fermented non‐alcoholic beverage, brewed across Malawi. The effect of boiling, fermentation and their interaction on the level of aflatoxins, the trends of aflatoxin reduction during brewing, and the distribution of aflatoxins between the solid and liquid phases of the beverage were studied using VICAM AflaTest immunoaffinity fluorometric assay. Fermenting and boiling *thobwa pre‐mix‐*, with initial aflatoxin content of 45–183 μg/kg, resulted in aflatoxin reduction of 47% (13–61 μg/kg) on average. Fermentation and boiling contributed about 20 and 33% aflatoxin reduction, respectively, but without interactive effect between the two factors. Fermenting the *thobwa* for 24 h led to further reduction of aflatoxins to about 37% of the initial content, and remainedconstant for up to 8 days. Thobwa is a popular beverage in Malawi which is consumed in large quantities by all gender categories including infants, therefore the presence of aflatoxins may constitute a significant health risk factor. This study highlights the need to use raw materials with low levels of aflatoxins for production of maize‐based non‐alcoholic beverages to ensure consumer safety.

## Graphical abstract

The tradtional brewing process reduces aflatoxin content in *thobwa*. However, substantial amount of aflatoxins still remains in the product. The use of the clean raw material is the only safest way for less contaminated *thobwa*.

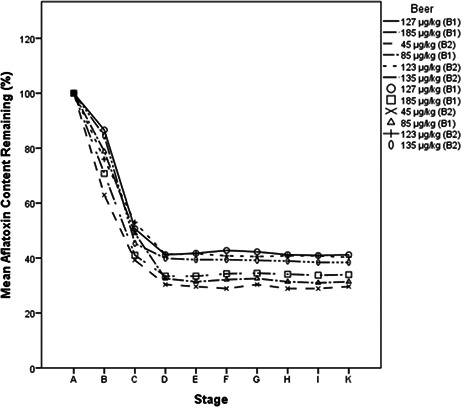

## 1 INTRODUCTION

*Thobwa* is a popular traditional fermented non‐alcoholic beverage prepared from various grains and their respective malts in Malawi. In central region of Malawi, *thobwa* is principally prepared by fermenting a brewed mixture of ground maize and malted maize. It is a light brown, relatively thick opaque gruel with a sweet‐acidic taste and is consumed by anyone including children. However, both maize and malted maize are prone to toxigenic fungal colonization and mycotoxin contamination including aflatoxins. Aflatoxins are acutely toxic, carcinogenic, mutagenic, teratogenic, and immunosuppressive, and are classified as group 1 human carcinogens (International Agency for Research on Cancer (IARC), 2002; Mohsenzadeh et al., 2016; Ostry et al., 2017).

The risk of aflatoxin contamination during malting may increase several folds due to increased moisture content especially under uncontrolled environments (Novellie & De Schaepdrijver, 1986; Schwarz et al., 1995; Wolf‐Hall, 2007) and high aflatoxin levels of up to 1020 μg/kg have been reported in malted maize in Malawi (Kenji et al., 2000). In central part of Malawi, some households reserve low grade shriveled maize for brewing *thobwa* and alcoholic beer for celebrations and income. Studies have indicated that alcoholic beer products prepared from low grade maize contain appreciable aflatoxin levels in the range of 90 ± 95 μg/kg (Matumba et al., 2014).

In general, brewing of alcoholic or non‐alcoholic beverages may involve malting, milling, mashing, lautering, wort boiling, wort cooling and fermentation followed by aging (lagering) and filtering (finishing) (Catarino & Mendes, 2011; Eblinger & Narzib, 2012; Embashu & Nantanga, 2019). Some of these brewing steps have been found to significantly reduce mycotoxin levels (Ezekiel et al., 2018; Lulamba et al., 2019a; Pascari et al., 2018). However, there is paucity of information on the  effect of the traditional brewing steps, used in the production of *thobwa,* on the on the reduction aflatoxin content in *thobwa*. Therefore, the present study reports on (i) trends of aflatoxin reduction during brewing (ii) effects of boiling and fermentation and their interaction on content of aflatoxin in *thobwa*; and (iii) the distribution of aflatoxin between the solid and liquid phases of the studied beverage.

## 2 MATERIALS AND METHODS

### 2.1 Maize and malt samples

Six visually moldy dried maize malts (6–10 kg each) were bought from a rural market in Lilongwe district (Malawi). Visually moldy maize spared for brewing fermented beverage was sourced from households in surrounding villages. Both maize and malt were separately ground into powder that passed through sieve # 20 (opening size = 0.85 mm) after which they were thoroughly mixed and used in *thobwa* preparation.

### 2.2 Study design

Three experiments were performed to understand the effect of various production steps of *thobwa* on aflatoxin content. The first experiment was performed to establish the trend of aflatoxin reduction during *thobwa* brewing. The second study was aimed at determining the effect of boiling, fermentation and their interaction on aflatoxin reduction in *thobwa*. The third (last) experiment was performed to determine the distribution of aflatoxins between the solid and liquid phases of *thobwa*.

#### 2.2.1 Determining the trend of aflatoxin reduction during brewing (experiment # 1)

**FIGURE 1:**
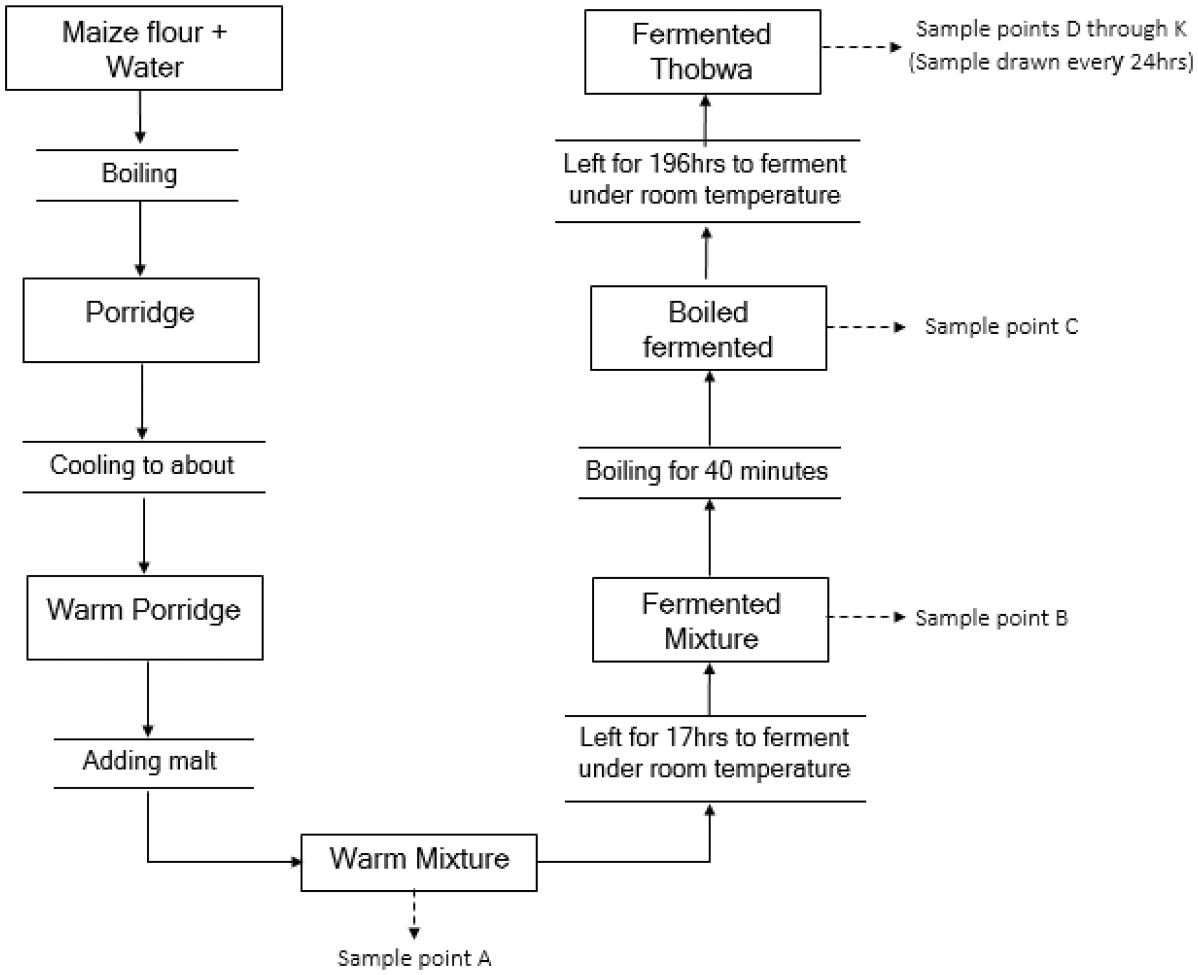
*Thobwa* brewing (experiment # 1).

Two sets of experienced *thobwa* brewers (women) were engaged to prepare *thobwa* at two different sites using a standardized procedure informed by preliminary trials and local expertise. The brewers were cautioned not to drink the *thobwa* as it contained high aflatoxin levels. The standardized procedure involved preparing a porridge from 15 liters of water and 4.5 kg of aflatoxin contaminated maize flour and boiling the mixture for 80 min. The porridge was then cooled to about 45°C followed by addition of 1.5 kg of ground malt and vigorous stirring. A sample was then drawn and immediately analyzed for aflatoxin concentration as described in section 2.3. Subsequently, samples were drawn at all critical stages of *thobwa* preparation outlined in Figure 1 and aflatoxin concentration were determined. Finally, aflatoxin levels were computed and expressed as percentage of the original mixture of the porridge and malt.

#### 2.2.2 Determining the effect of boiling and fermentation on aflatoxin in *thobwa* (experiment # 2)

The second experiment (2 × 2 factorial design), aimed at determining the effect of boiling, fermentation and their interaction on aflatoxins reduction in *thobwa* was superimposed onto Experiment #1 at one brewer site. The experiment investigated two variables (boiling and fermentation) at two levels coded ‘no’ (absent) or ‘yes’ (present). To realize a full factorial design, a non‐traditional boiling step was introduced immediately after mixing porridge and ground malt (Sampling point A; Figure 1) to obtain sampling point herein referred to as point X (Table 1) to elucidate effects of boiling alone. Thus, proportion of aflatoxin remaining in mixtures/*thobwa* at sampling points A, B, C (all displayed in Figure 1) and sampling point X (the non‐traditional step) were statistically analyzed with the three mixtures/*thobwa* at ‘brewer set 1’ serving as replications.

**TABLE 1:** Factorial design matrix for the boiling and fermentation of beer.

Boiling	No	Yes
No	No fermentation No boiling (sampling point A in Figure 1)	Fermentation No boiling (sampling point B in Figure 1)
Yes	No fermentation Boiling (sampling point X, a non‐traditional step)	Fermentation and boiling (sampling point C in Figure 1)

#### 2.2.3 Determining the distribution of aflatoxin in *thobwa* (experiment #3)

Like Experiment #2, Experiment #3, which aimed at determining the distribution of aflatoxin between the solid and liquid phases of the *thobwa*, was superimposed onto Experiment 1. In this regard, sub‐samples of mixtures/*thobwa* at sampling points A through to E (Figure 1) were filtered using VICAM fluted filter paper # 1289 (Whatman 2V, Whatman Middlex) and the filtrates analyzed for aflatoxin content using immunoaffinity fluorometric analysis as described in section 2.3. The aflatoxin levels of the filtrate derived from sampling point A, B, C, D and E were expressed as proportion of the whole mixture at each respective sampling point and the whole mixture at point A (mixture of porridge and ground malt).

### 2.3 Total aflatoxins determination

Extraction and clean‐up of aflatoxin from sample test portions were performed using AflaTest® immuno‐affinity fluorometric procedures for popcorn according to the manufacturer's instruction with slight modification (VICAM, 2014). Aflatoxin was extracted from the *thobwa* and filtrate test portions (50 g test portion +5 g NaCl) using 200 mL of HPLC grade methanol (100%) and blended at high speed for 2 min. The triturated mixture was filtered through Sartorius grade 1289 fluted filter paper to remove particulate matter. A 10 mL volume of the filtered extract was diluted using 40 mL distilled water, thoroughly mixed and filtered through a glass‐fiber filter paper of 1.5 μm diameter. A 20 mL (1 g sample equivalent) of the diluted extract was passed through AflaTest® immuno‐affinity column at a flow rate of about 1 drop/second. The column was washed twice using (10 mL) distilled water at a rate of 1 drop/second to remove maize intrinsic compounds and finally the aflatoxins were selectively eluted with 1 mL of 100% HPLC grade methanol into a glass cuvette. 1 mL of AflaTest® developer (developer/water of 1 + 9, v/v) was added to eluate in the cuvette and mixed using a vortex and absorbance read using a calibrated VICAM Series‐4EX Fluorometer, Milford, MA, USA. The method had limit of detection of 1 μg/kg and was validated using artificially contaminated *thobwa* at a concentration of 25 μg/kg aflatoxin B_1_ (Sigma Aldrich) which yielded recovery of 95% with relative standard deviation (RSD) of 2%.

### 2.4 Statistical analysis

The differences among means were analyzed by one‐way analysis of variance (ANOVA). Results showing significant differences were subjected to post‐hoc Tukey's test. The linear regression analysis was performed to study the effects of the boiling, fermentation and their interaction on aflatoxin reduction. The level of confidence required for significance was set at *p* ≤ .05 for all statistical analyses. These analyses performed using XLSTAT (version 19.01; Addinsoft, New York) and SPSS version 21 (IBM Corp, Armonk, New York).

## 3 RESULTS

### 3.1 Reduction of aflatoxin levels during brewing

**FIGURE 2:**
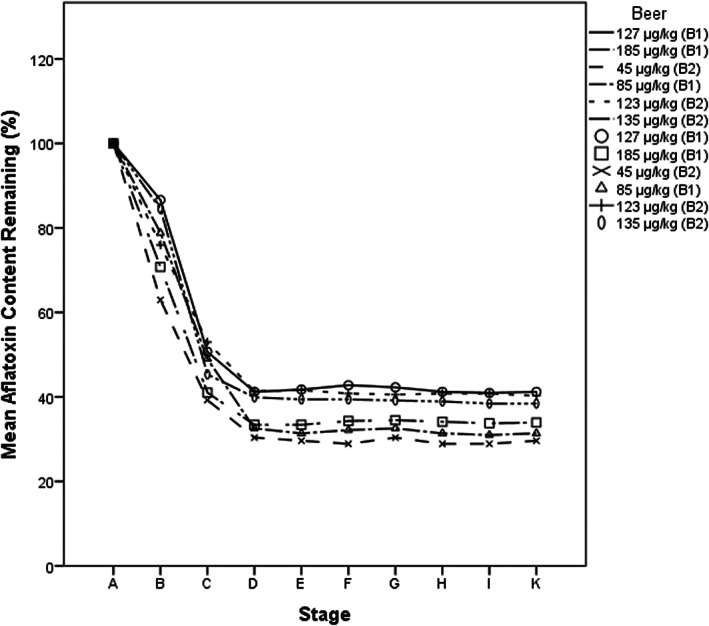
Fate of aflatoxin during brewing and storage of *Thobwa*. ‘A’ represents, warm mixture of porridge and malt on the onset of the brewing process; ‘B’ is 17‐h fermented mixture before boiling; ‘C’ is 17‐h fermented mixture (beverage) after boiling for 40 min, ‘D’ through to ‘K’ are fermented beverage 1–8 days after boiling, respectively. ‘B1’ represents beverage samples from brewers set 1 and ‘B2’ represents beer samples from brewers set 2.

The reduction of aflatoxin content in six *thobwa* samples whose original concentrations ranged from 45 to 183 μg/kg is graphically presented in Figure 2. Aflatoxins were detected at levels of about eighty percent (77 ± 9%) of the initial aflatoxin levels after 17 h of fermentation (Stage B). Boiling the 17‐h fermented mixtures for 40 min significantly (*p* < .05) reduced aflatoxins content to 46 ± 6% of the original levels (Stage C). Testing the finished product (*thobwa*) after 24 h of storage showed further reduction of aflatoxins and only 36 ± 5% of the very original aflatoxin concentrations (16–66 μg/kg) were detected in the *thobwa* (Stage D). Subsequently, aflatoxin concentration remained constant for 8 days of evaluation (Stages D–K).

### 3.2 Effect of boiling and fermentation on aflatoxin

**FIGURE 3:**
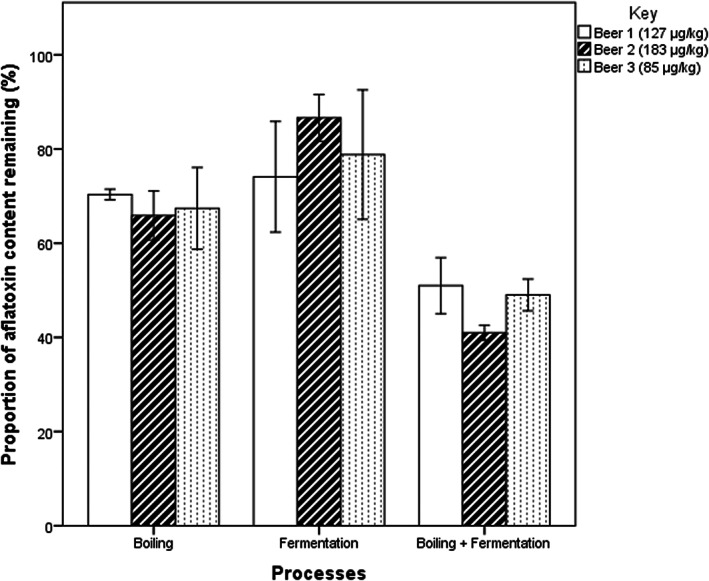
The effect of boiling, fermentation and combination thereof on aflatoxin content in three *Thobwa* samples.

The results of effect of boiling, fermentation and their combination on aflatoxin levels in three *thobwa* samples are displayed in Figure 3. Boiling *thobwa* mixtures prior to fermentation (not a normal procedure, only performed in this experiment to elucidate singular effect of boiling) caused a mean reduction of 33% in aflatoxin levels. Fermenting non‐boiled mixtures (normal procedure) caused about 20% reduction of aflatoxins.

The linear regression results displayed in Table 2 clearly demonstrated that there was no interaction between boiling and fermenting the *thobwa* mixtures as regards reduction of aflatoxins as there was no noticeable significant surplus effect of combining the two processes.

**TABLE 2:** Effect of fermentation and boiling on percentage aflatoxin content in *Thobwa* during brewing process.

Source	Value	Standard error	*t*	Pr > |t|	Lower bound (95%)	Upper bound (95%)
Intercept	99.417	2.212	44.952	<0.0001	94.414	104.420
No boiling	0.000	0.000				
Boiling	−32.167	2.554	−12.596	<0.0001	−37.944	−26.390
No fermentation	0.000	0.000				
Fermentation	−19.167	2.554	−7.505	<0.0001	−24.944	−13.390
No fermentation * No boiling	0.000	0.000				
Fermentation * No boiling	0.000	0.000				
No fermentation * Boiling	0.000	0.000				
Fermentation * Boiling	0.000	0.000				

*Note*: Model: Aflatoxin Remaining (%) = 99.417−32.167 * Boiling −19.167 * Fermentation.

### 3.3 The proportion of aflatoxins present in liquid phase of the *thobwa*

**FIGURE 4:**
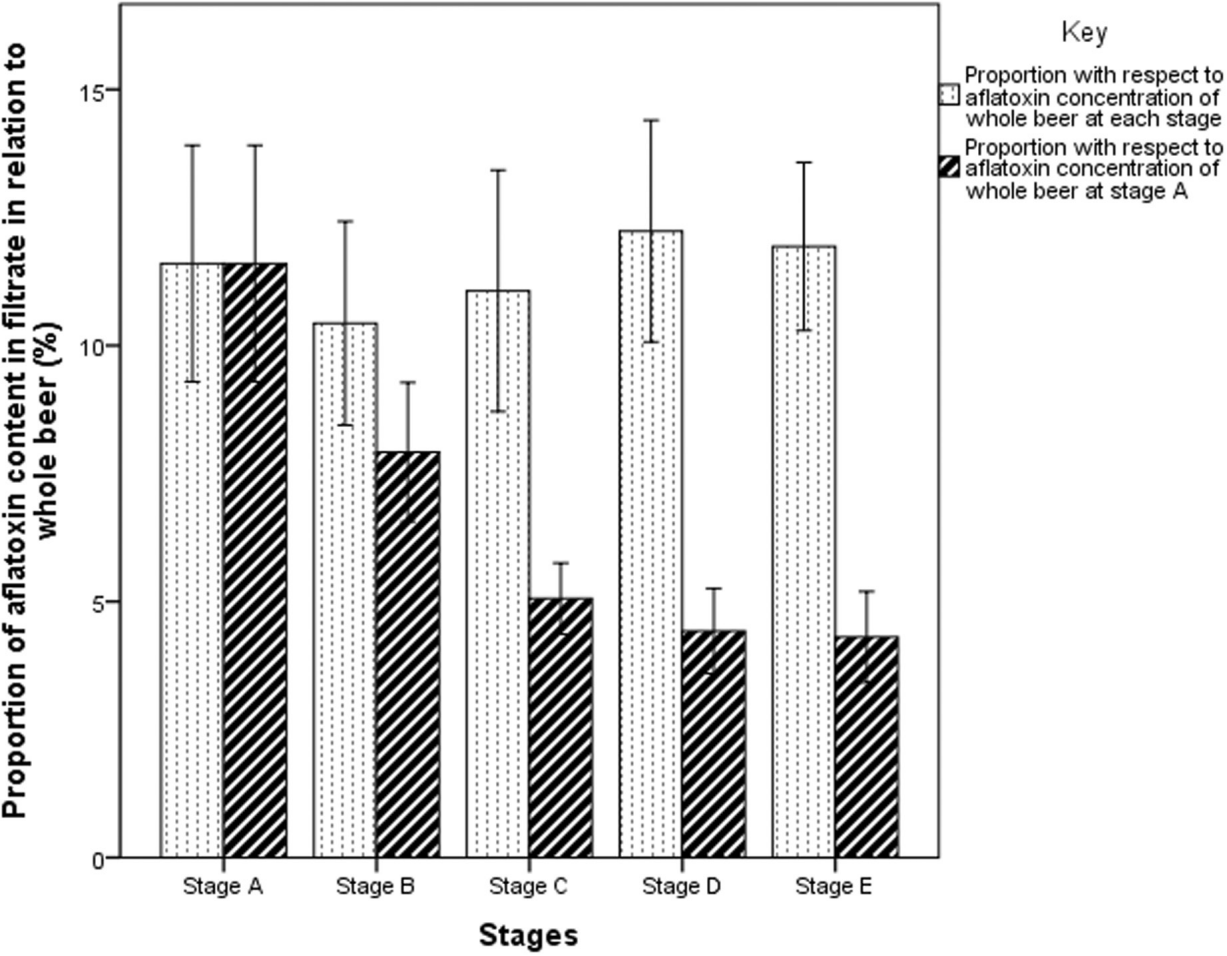
Fate of aflatoxin content in filtrate during brewing and storage of the *Thobwa*. ‘A’ represents, warm mixture of porridge and malt on the onset of the brewing process; ‘B’ is 17‐h fermented mixture before boiling; ‘C’ is 17‐h fermented mixture (beverage) after boiling for 40 min, ‘D’ and ‘E’ are fermented beverage on first and second day after boiling respectively.

The proportions of aflatoxin content detected in liquid phase (filtrate) of the *thobwa* samples with respect to the whole *thobwa* mixtures during each of the five stages of brewing are plotted in Figure 4. The proportion of aflatoxin in the filtrate remained statistically (*p* < .05) constant (10.4–12.2%) throughout the brewing process. But if considered with respect to the original concentration of aflatoxins in the whole mixture (porridge plus malt) measured at the onset of the experiment (Stage A), the proportions of aflatoxin content in liquid phase declined following 17‐h fermentation process (Stage B) and the boiling of the fermented mixtures (Stage C).

## 4 DISCUSSION

Aflatoxins are potent hepatotoxic and carcinogenic mycotoxins, ubiquitous in maize grains and maize malts in Malawi (Kenji et al., 2000; Matumba et al., 2013). Therefore, the deep understanding of their (aflatoxins) degradation or stability during brewing of *thobwa* is necessary for the development of feasible approaches in minimizing consumer risks. *Thobwa* is a popular beverage in Malawi which is consumed in large quantities by all gender categories including infants, therefore the aflatoxin presence may constitute a significant health risk factor.

The present study has for the first time broadly examined the effects of fermentation and boiling on aflatoxin levels during brewing of *thobwa*. The results indicate that both fermentation and boiling of pre‐*thobwa* mixtures result in significant aflatoxin reduction with boiling having greater effect compared fermentation. Although this study did not investigate the mechanism of aflatoxin reduction, it can be postulated based on the findings from other studies that fermentation led to irreversible microbial binding/conjugation (Hamad et al., 2017) and/or enzymatic transformations of the aflatoxins thereby making them escape analysis of its parent forms (Adebo et al., 2017; Wang et al., 2018). Likewise, it can be postulated from existing literature that boiling may have also led to hydrolytic opening of the lactone ring in the aflatoxin followed by heat‐induced decarboxylation, leading to loss of the methoxy group in the aromatic ring (Farah et al., 1983; Rustom et al., 1993).

Thermal treatments of food containing mycotoxins have been reported to yield metabolites that are as toxic as their parent mycotoxins or even worse (BenáTrivedi, 1993; Dombrink‐Kurtzman et al., 2000; Voss & Snook, 2010). However, for aflatoxins, several toxicological studies using cytotoxicity assays on mammalian cell lines or live animal subjects have demonstrated that aqueous boiling and fermentation produce aflatoxin degradation products with reduced toxicity and carcinogenicity (Aiko et al., 2015; Liu et al., 2017). For instance, aflatoxin B2a, degradation product of bacterial conversion of aflatoxin B1, has shown to exhibit lower DNA‐binding capacity than its parent mycotoxin (aflatoxin B1). Therefore, the reduction of the aflatoxin levels observed in the present study suggests that brewing of *thobwa* had comparatively lowered toxicity than their original mixtures. However, it is noteworthy that in the present experiment, *thobwa* samples were not completely safe for human consumption since significant proportions (36–46%) of the parent aflatoxin compound were detected. Thus, while brewing may significantly improve the safety of beers, it is necessary to ensure that the raw materials (maize and malts) contain as low aflatoxin as possible to guarantee consumer safety particularly among infants and children.

The stability of aflatoxins during brewing witnessed in this study is of great importance in Malawi considering the common practice of reserving low‐grade grains (with high aflatoxin levels) for preparation of *thobwa* for celebratory and rituals functions by majority of the households. This practice, likely, exposes *thobwa* consumers to high dietary aflatoxin levels as already demonstrated by earlier studies (Matumba et al., 2014). Under the present investigation, it was observed that fermentation alone was not sufficient to reduce the aflatoxin in the *thobwa*. Seventeen hours of natural fermentation of the pre‐mix‐*thobwa* only degraded 20% of the toxins. Contrarily, 70–100% degradation of aflatoxins was observed when bacteria starters are added (Shu et al., 2018; Wacoo et al., 2019). However, in Malawi, traditional preparation of *thobwa* does not involve addition of culture starters although it is becoming a popular practice among alcoholic brewers within the area under study. Given the many nutritional benefits associated with probiotics (Nagpal et al., 2012) and the aflatoxin degradation capability of some strains, promoting a culture of probiotic enrichment of *thobwa* seems plausible.

This study is unique in that it was carefully designed to allow an investigation of the singular effect of boiling pre‐mix‐*thobwa* on aflatoxin degradation in *thobwa* (final product) through the use of full factorial design and regression analysis. Under normal practice, after mixing porridge and ground malt, the mixture is left overnight to ferment. However, the introduction of a non‐traditional boiling step after mixing porridge and ground malt enabled the elucidation of the singular effect of boiling *thobwa*. The appreciable (31%) degradation of aflatoxin arising from the single effect of boiling witnessed in the present is of significance. Since its discovery in the 1960s, aflatoxins have been regarded as thermally stable (WHO (World Health Organization), 1998), consequently not much work has been performed to investigate conditions that may enhance its degradation. While aflatoxins might indeed be quite stable to dry heating, they seem to be unstable under moist heat and the present findings further reaffirms such phenomenon (Asghar, 2011; Samarajeewa et al., 1990).

The comparatively lower aflatoxin levels detected in filtrate of the beers partly explains the disparity between aflatoxin levels recorded in opaque fermented beverages of Africa and clear beers brewed in industrialized world (Lulamba et al., 2019b). While the absence of aflatoxins in industrial beers could be primarily due the low aflatoxin levels in the raw materials used, the processing effect cannot be ruled out. Aflatoxins are poorly soluble in water (International Agency for Research on Cancer (IARC), 2002) consequently during the lautering (a process of separating clear liquid wort from residual grains employed in brewing clear industrial beers), aflatoxins predominately remain in spent grains (Pascari et al., 2018; Rodrigues, & Chin, 2012), thus making clear beers comparatively safer from aflatoxin contamination compared to opaque beers. On the other hand, the non‐uniform distribution of aflatoxin between the filtrate and the solids observed in this study presents a sampling challenge. If an analyst sampled a clear liquid part of the beverage (after settling), under‐reporting of aflatoxin is likely. Conversely, if a sample was to be drawn from the bottom part or after removal of most of the liquid proportion, over reporting is likely.

The study was modeled based on a recipe mainly used in the central region of Malawi which differs from that of other regions within the country. Even within the central region, there are likely to be some variations particularly in terms of cooking time, cooking temperatures, water‐matrix ratio, fermentation periods and fermentation temperatures. In the present study, all these parameters were held constant except for the cooking temperatures and the fermentation temperatures which were beyond the control of the researchers. Moreover, it is likely that the type and concentration of microbiota would differ from one environment to the other and yet the present study was limited to one location (Lilongwe) and one season. These factors may affect aflatoxin degradation outcomes. Nonetheless, being the first study of its kind, the current results provide useful insights that would inform the design of future comprehensive studies on fate of aflatoxin during brewing of *thobwa*.

## 5 CONCLUSION

While there is substantial reduction of aflatoxins during preparation of *thobwa*, a significant proportion of aflatoxins may still remain in the final product. These findings highlight the need to use aflatoxin safe raw maize grains and malt during preparation of the beverage if safety of consumers is to be guaranteed (Matumba et al., 2021). Further, there is need to develop practical strategies to hinder fungal proliferation during malting such as the use of herbs (Abd El‐Aziz et al., 2012). The study has provided some insights, however, it necessary to identify and quantify aflatoxin thermal degradation or matrix bound products that arise during the beverage preparation and assess their toxicological safety.

## CONFLICT OF INTEREST STATEMENT

We declare that we have no conflict of interest.

## Data Availability

The data that support the findings of the study are available from the corresponding author upon reasonable request.
